# Preventive Potential of Resveratrol in Carcinogen-Induced Rat Thyroid Tumorigenesis

**DOI:** 10.3390/nu10030279

**Published:** 2018-02-28

**Authors:** Xu Zheng, Bin Jia, Xue Song, Qing-You Kong, Mo-Li Wu, Ze-Wen Qiu, Hong Li, Jia Liu

**Affiliations:** 1 Liaoning Laboratory of Cancer Genetics and Epigenetics and Department of Cell Biology, College of Basic Medical Sciences, Dalian Medical University, Dalian 116044, China; dyzhengxu@yeah.net (X.Z.); jiabinab@aliyun.com (B.J.); songxue0214@163.com (X.S.); kqydl@sina.com (Q.-Y.K.); moliwusx@sina.com (M.-L.W.); lihongmcn@dlmedu.edu.cn (H.L.); 2 Laboratory Animal Center, Dalian Medical University, Dalian 116044, China; qiuzewens@163.com

**Keywords:** thyroid tumorigenesis, resveratrol, chemoprevention

## Abstract

Thyroid cancer (TC) is the most common endocrine malignancy without reliable preventive agent. Resveratrol possesses in vitro anti-TC activities; while its effect(s) on thyroid tumorigenesis remains unknown. This study aims to address this issue using DEN/MNU/DHPN-induced rat carcinogenesis model. 50 male Sprague-Dawley rats were separated into four groups as Group-1 (5 rats); normally fed; Group-2 (15 rats); DEN/MNU/DHPN treatment only; Group-3 (15 rats) and -4 (15 rats); DEN/MNU/DHPN treatment; followed by resveratrol intragastric (IG) injection and intraperitoneal (IP) injection; respectively; in two-day intervals for 30 weeks. The results revealed that the average resveratrol concentration in thyroid tissues was 1.278 ± 0.419 nmol/g in IG group and 1.752 ± 0.398 nmol/g in IP group. The final body weights of Group-3 and Group-4 were lighter than that (*p* > 0.05) of Group-1; but heavier than Group-2 (*p* < 0.05). TC-related lesions (hyperplasia and adenomas) were found in 53.3% of Group-2; 33.3% Group-3 and 26.7% Group-4. Lower serum carcino-embryonic antigen (CEA) and thyroglobulin (Tg) levels; down-regulated expression of IL-6 and cyclooxygenase-2 (COX-2); reduction of NF-κB/p65 nuclear translocation; and elevated IkB*α* expression were found in the thyroid tissues of Group-3 and Group-4 in comparison with that of Group-2. These results demonstrate that IG and IP administered resveratrol efficiently reduces the frequency and severity of DEN/MNU/DHPN-caused TC-related lesions and would be of values in thyroid tumor prevention.

## 1. Introduction

Thyroid cancer (TC) is one of the commonest endocrinal malignancies in worldwide [1]. For instance, Chinese TC incidence keeps increasing in the last 30 years, irrespective to the improved health care and popularization of iodized salt consumption [2]. Many factors are supposed to be related with thyroid carcinogenesis such as genetic mutations, environmental factors, life style, obesity, and ionizing radiation, while their implications in TC prevention and treatment remain uncertain [3,4,5,6]. Thyroidectomy assisted by radioactive iodine therapy has become the mainstream of TC treatment and achieved promising therapeutic outcome [7]. However, the post-operation complications such as parathyroid and recurrent laryngeal nerve, laryngeal nerve injury, seriously affect the quality of life of patients. In addition, the life-long thyroid-hormone supplementation or replacement therapy is a heavy financial burden to TC patients and society [8]; secondary risk of malignant tumors and cancer recurrence of the diagnosis of patients also bring a psychological burden [9]. In this context, it would be more valuable to explore the safer and reliable approach to prevent thyroid tumor formation.

Resveratrol in the chemical name of 3,4′,5-trihydroxystilbene has a wide range of health benefits for the coronary, nervous, liver, and cardiovascular systems [10]. Resveratrol also exerts suppressive effects on many types of cancers including thyroid cancers by inducing differentiation and apoptosis via inhibiting cancer-related gene expression and cancer-associated signaling [11]. More importantly, the anticancer doses of resveratrol are not harmful to normal cells and tissues [12]. Our recent studies reveal that resveratrol effectively suppresses in vitro growth and overcomes the retinoic acid resistance of anaplastic TC cells [13], suggesting the potential values of this nontoxic compound in TC prevention and treatment if its anti-TC effects can be further confirmed in vivo. 

**Figure 1:**
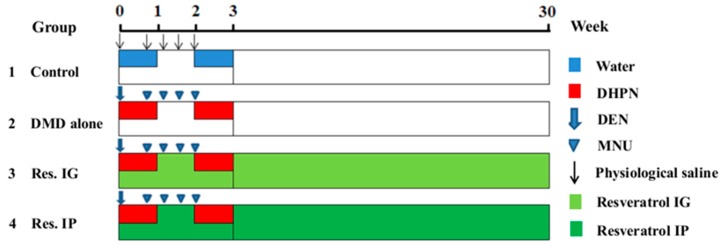
Experiment protocol for DMD-induced rat carcinogenic model. Rats in G2–G4 were sequentially treated with DEN (100 mg/kg body weight, IP, single dose), MNU (20 mg/kg body weight, IP, four times, on days 5, 8, 11, 14) and DHPN (0.1% in the drinking water, during weeks 1 and 3). Rats in G3 received resveratrol (20 mg/kg by body weight) via intragastric route (IG) in two day intervals for 30 weeks. Rats in G4 received resveratrol (20 mg/kg body weight) via intraperitoneal route (IP) in two day intervals for 30 weeks. The rats in G1 were given vehicle alone.

Chemoprevention aims to use natural or synthetic compounds to intervene against development of early precancerous stages and tumor formation in stepwise carcinogenesis [14]. Carcinogen-induced animal tumor models are indispensable tools for investigating the new therapeutic and chemopreventive approaches for cancers [15]. DMD is constituted by three different genotoxic carcinogens: diethylnitrosamine (DEN), *N*-methyl-*N*-nitrosourea (MNU) and Dihydroxy-di-*N*-propyl-nitrosamine (DHPN). DEN is a specific carcinogen of liver [16], DHPN is main induction of lung and thyroid carcinogenesis [17,18], and MNU targeted for breast and gastrointestinal tract [19,20]. Based on the initiation-promotion concept, this DMD carcinogenic model (shown in Figure 1) was adopted to evaluate the preventive potential of resveratrol on stepwise thyroid carcinogenesis [21].

## 2. Materials and Methods

### 2.1. Animals

Five-week-old male Sprague-Dawley (SD) rats were obtained from Animal Center of Dalian Medical University. They were housed in polycarbonate cages with hard wood chips at a temperature of 23 ± 2 °C and a humidity of 55 ± 5% with a 12 light/dark cycle. Diet and drinking water were available ad libitum. After a one-week acclimation period, the animals were subjected to the treatments. All of the animal experimental protocols were approved by the Committee of Animal Care and Welfare, Dalian Medical University. All work involving experimental animals was performed in full compliance with NIH (National Institutes of Health) Guidelines for the Care and Use of Laboratory Animals. The animal experiments were performed under chloralhydrate anesthesia and all efforts were made to minimize suffering.

### 2.2. Chemicals

DEN (Diethylnitrosamine, CAS No. 55-18-5), MNU (*N*-methyl-*N*-nitrosourea, CAS No. 684-93-5) and DHPN (Dihydroxy-di-*N*-propyl-nitrosamine, CAS No. 53609-64-6) were purchased from J & K Scientific Ltd., (Beijing, China). Resveratrol, dimethyl sulfoxide (DMSO), HPLC-grade acetonitrile, methanol, acetic acid, and 1,8-dihydroxyanthraquinone were purchased from Sigma-Aldrich (St. Louis, MO, USA).

### 2.3. Experimental Design

As shown in Figure 1, the experimental procedure was undertaken when the rats were six weeks old. Fifty male SD rats were randomly divided into four groups. Animals of groups 2–4 were subjected to the DMD initiation, consisting of DEN (dissolved in physiological saline solution, 100 mg/kg by weight, a single intraperitoneal injection, at the commencement of the experiment), MNU (dissolved in citrate-buffered solution pH 6.0, 20 mg/kg by weight, intraperitoneal injection, four times at days 5, 8, 11, and 14), and DHPN (0.1% in the drinking water for two weeks during weeks 1 and 3). Non-initiation controls (Group-1) were given vehicle in the drinking water instead of the carcinogen injections. The rats in Group-3 and Group-4 were treated by resveratrol in the dose of 20 mg/kg body weight via intragastric and intraperitoneal routes in two day intervals for 30 weeks. Rats in Group-1 without DMD and resveratrol treatment were cited as control. The animals in each group were sacrificed under ether anaesthesia at the 30th week and their body weights and relative organ weights were recorded. Portions of the tumor-containing tissues were snap frozen with evaporated liquid nitrogen (−80 °C) for frozen sectioning and protein preparation; the remaining tissue was fixed in 10% formalin, embedded into paraffin and sectioned for morphological and immunohistochemical examinations. The serum samples were obtained from the inferior vena cava blood. Blood samples for serum isolation (1 mL) were maintained for 15–20 min at room temperature after extraction to allow for clotting and then centrifuged at 2000 rpm for 10 min. The supernatants were stored at −80 °C until use.

### 2.4. Elucidation of Resveratrol Availability in Thyroid Tissues and ATC Cells

#### 2.4.1. Sample Collection and Treatments

Ten rats were randomized into two groups: Group-1, treated with resveratrol intragastric ingestion; and, Group-2, treated with resveratrol intraperitoneal injection. 30 min after 20 mg/kg resveratrol intragastric or intraperitoneal administration, the rats were painlessly sacrificed by cervical spinal dislocation and their thyroids were immediately collected. Briefly, thyroid tissues were dissected on an ice bed, wrapped in aluminum foil, snap frozen in liquid nitrogen and stored at –80 °C until use. To evaluate of the efficacy of drug uptake, 100 μM resveratrol-treated human anaplastic thyroid cancer THJ-16T cells [13] were employed as effective control. THJ-16T cells was cultured in RPMI 1640 medium (Gibco, Grand Island, NY, USA) supplemented with 5% fetal bovine serum (Gibco, Grand Island, NY, USA) at 37 °C in a humidified atmosphere containing 95% air and 5% CO_2_ [22]. Resveratrol was dissolved in DMSO to a stock concentration of 100 mM, and stored in darkness at −20 °C. THJ-16T cells were treated with 100 μM resveratrol for 60 min and then collected. The cells and thyroid tissues without resveratrol treatment were cited as background control. 

#### 2.4.2. Sample Preparation and HPLC Analyses

Sample tissues and cells were extracted with 416 μL methanol and 84 μL 1,8-dihydroxyanthraquinone (internal standard, IS, 200 μg/mL) in a centrifuge tube. The tissue homogenates were centrifuged at 12,000 rpm for 10 min at 4 °C and the supernatant was transferred to a clean tube. The combined organic solvent of the supernatants was evaporated to a final volume of 400 μL, and subsequently placed in a sealed amber vial for HPLC analysis [23]. To improve the sensitivity and precision of quantification, we purified the samples and cited 1,8-dihydroxyanthraquinone (Sigma-Aldrich, St. Louis, MO, USA) as the internal standard and trans-resveratrol (Sigma-Aldrich) as the standard for drawing a standard curve by the methods described elsewhere [24].

### 2.5. ELISA Assay for Thyroid Cancer-Related Markers

Serum CEA and Tg levels were determined using a ELISA kits (Lengton Bioscience Co., Ltd., Shanghai, China). Briefly, serum samples were incubated in 96-well microplates coated with anti-mouse primary antibodies against CEA or Tg. Samples were developed with horseradish peroxidase-conjugated secondary antibodies. After adding the substrate and stop solution, the plates were read on a microplate reader (Thermo Fisher Scientific, Waltham, MA, USA) using a test wavelength of 450 nm. The results were calculated based on the absorbance of complex cytokines–antibodies. The concentrations were obtained from model curves with 0.1 and 0.2 ng/mL detection limits for Tg and CEA, respectively.

### 2.6. Histological Staining and Examination

Paraffin-embedded thyroid as well as liver, colon, lung tissues of the four experimental groups rats were sliced into 5 μm sections and then subjected to hematoxylin and eosin (HE) staining for histological examination. Thyroid lesions were pathologically classified into focal follicular cell hyperplasias (FFCHs), adenomas, and carcinomas, according to the published criteria [25]. Pathological sections were double-blindly read by two pathologists. 

### 2.7. Immunohistochemical Staining

Immunohistochemical (IHC) staining was conducted using DAB substrate kit (ZSGB-BIO, Beijing, China) and the antibodies against Ki-67 (rabbit IgG, 1:150 dilution; Proteintech Group, Inc., Rosemont, IL, USA), PCNA, TTF-1 and Tg (Mouse monoclonal IgG, 1:50 dilution; Santa Cruz Biotech, Santa Cruz, CA, USA), NF-κB, COX-2 and IL-6 (Mouse monoclonal IgG, 1:50 dilution; Santa Cruz Biotech, Santa Cruz, CA, USA) and IkBα (1:100 from Cell Signaling, Cat., Danvers, MA, USA), respectively [26]. The results were evaluated according to the labeling intensity and scored as negative (−), weakly positive (+), moderately positive (++), and strongly positive (+++).

### 2.8. Western Blot Analysis

For Western blotting, total cellular proteins were prepared from the thyroid tissues by the method described previously [27]. Fifty micrograms of sample protein was separated with 12% SDS/PAGE, and transferred to polyvinylidene difluoride membrane (Amersham, Buckinghamshire, UK). The membrane was blocked with 5% skimmed milk in NaCl/Tris-T (10 mM Tris-Cl, pH 8.0, 150 mM NaCl, and 0.5% Tween-20) at 4 °C overnight. It was rinsed three times (10 min each) with NaCl/Tris-T, and this was followed by 3 h of incubation with the same first antibodies that were used in immunohistochemical staining in the appropriate concentrations (IL-6, 1:500; COX-2, 1:500; NF-κB/p65, 1:500; β-actin, 1:3000, and IkBα, 1:1000) and 1 h of incubation with horseradish peroxidase-conjugated anti-rat IgG (Zymed Laboratories, San Francisco, CA, USA). Immunolabeling was detected with an enhanced chemiluminescence system (Roche, Mannheim, Germany), and visualized with the UVP Bio-spectrum Imaging System (UVP, Upland, CA, USA). β-actin was used as the internal quantitative control in densitometry analyses.

### 2.9. Statistical Analysis

Statistical analyses were completed with SPSS statistical package 16.0 (Chicago, IL, USA). Data shown are mean ± SD values. The incidences of pathological lesions between groups were determined by use of Fisher’s exact probability tests. Differences of the mean values were analyzed using one-way ANOVA (LSD *t*-test) and Student’s *t*-test. The criterion for the differences was considered to be significant at *p* < 0.05.

## 3. Results

### 3.1. Sufficient Resveratrol Availability in Thyroids

**Figure 2:**
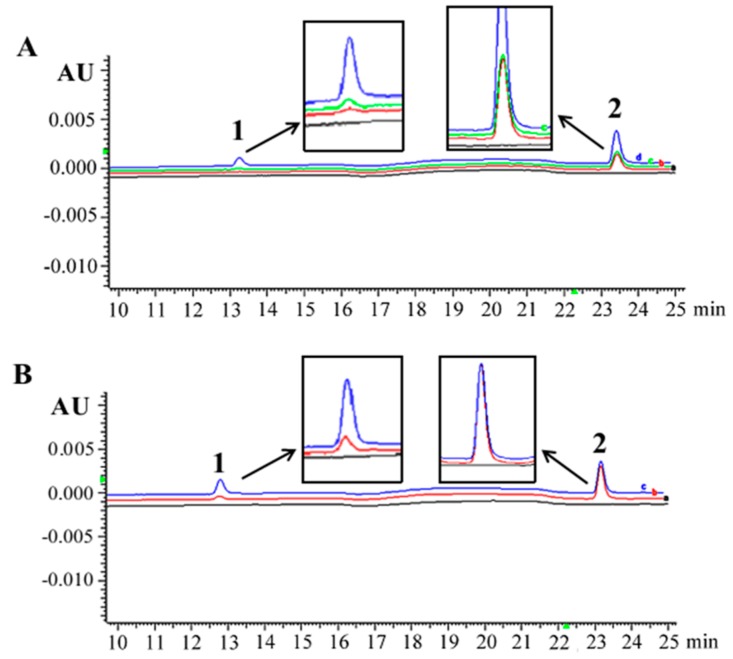
Representative HPLC/DAD analysis of resveratrol in rat thyroid tissue samples and THJ-16T thyroid cancer cells. (**A**) HPLC/DAD chromatograms of normal rat thyroid tissue (black line), thyroid tissue obtained from the rat 30 min after 20 mg/kg resveratrol injection through IG (red line) and IP (green line), normal rat thyroid tissue spiked with 1.0 μM resveratrol and internal standard/IS (blue line); (**B**) HPLC/DAD chromatogram of control cell lysates (black line), cells treated with 100 μM resveratrol for 60 min (red line), and control cell lysates spiked with 1.6 μM resveratrol and internal standard/IS (blue line). Peaks: 1. trans-resveratrol; 2. 1,8-dihydroxyanthraquinone (IS).

HPLC/DAD analysis of resveratrol in rat thyroid tissue samples was conducted 30 min after injection with 20 mg/kg resveratrol by IG or IP. As shown in Figure 2A, the average resveratrol concentration of thyroid tissues was 1.278 ± 0.419 nmol/g (0.256 μM) in IG group and 1.752 ± 0.398 nmol/g (0.351 μM) in IP group (Figure 2A). The intracellular concentration of resveratrol in 5 × 10^6^ THJ-16T cells was 0.362 ± 0.126 μM after 100 μM resveratrol treatments for 60 min (Figure 2B). Only one peak corresponding to trans-resveratrol was detected in both the thyroid tissues and THJ-16T cells extracts (Figure 2A,B). Cellular resveratrol uptake of 100 μM resveratrol-treated THJ-16T cells was about 41.4% and 3.1% higher than that of thyroid tissues treated by resveratrol through IG and IP routes, respectively.

### 3.2. Safety of Long-Term Resveratrol Treatment

No rat is dead during 30 weeks of repeated resveratrol treatment in two-day intervals. As shown in Table 1, the average final body weights of the resveratrol IP group (591.3 ± 38.4 g) and resveratrol IG group (580.5 ± 37.7 g) are higher than that of Group-2 (549.1 ± 42.1 g) with statistically significance (*p* < 0.05). No significant difference of the final body weights and relative weights of the thyroids, liver, lungs, kidneys, and spleen are established either between resveratrol IP group and resveratrol IG group or the control group and the two resveratrol-treated groups (*p* > 0.05).

### 3.3. Resveratrol Alleviated Thyroid Tissue Lesions

**Figure 3:**
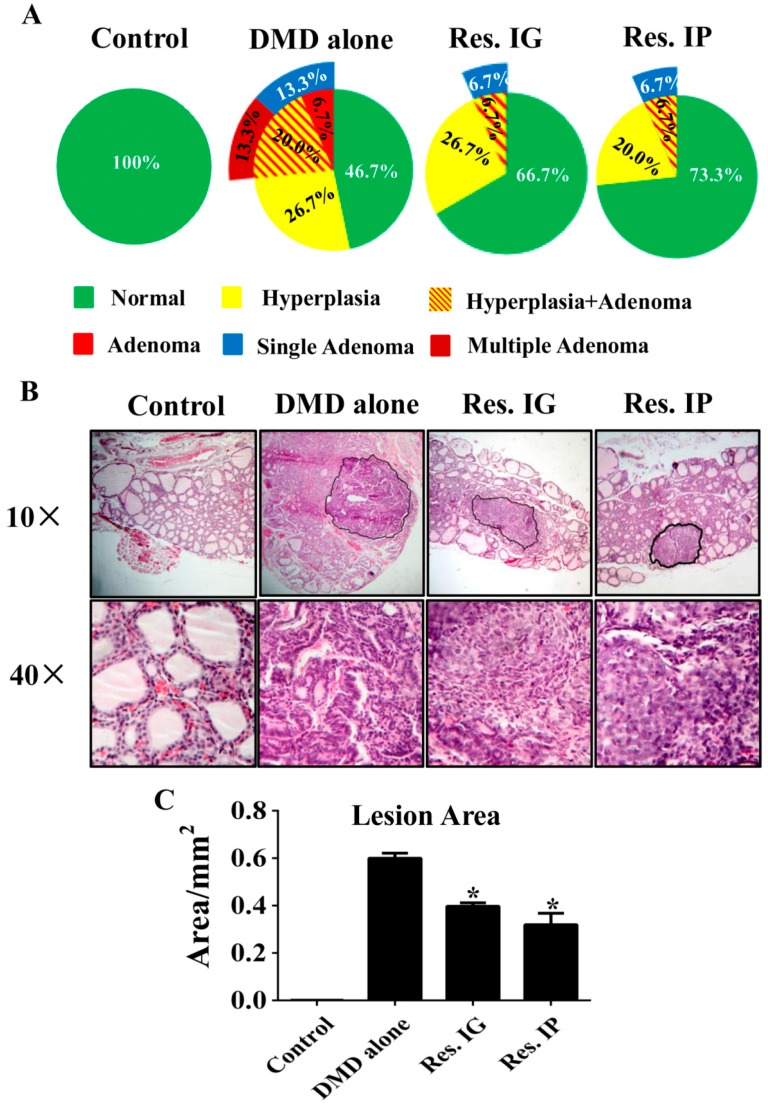
The incidences and areas of DMD-caused thyroid lesions in the four experimental groups. (**A**) Incidences and severity of pathological alterations in the thyroids; (**B**) Representative morphological findings from the thyroid tissues of the four experimental groups. The regions with morphlogical alteration are defined for area calculation; (**C**) The average areas of the thyroid lesions (hyperplasia/adenomas) were determined using Adobe Photoshop CS5. *, *p* < 0.05 in comparison with DMD alone group.

Histopathological examination was performed on each of the thyroid samples of the experimental groups. As shown in Figure 3A, no histological alteration was observed in the specimens of normally fed rats. Preneoplastic lesions (hyperplasia and/or adenomas) could be found in the DMD alone group, resveratrol IG group and resveratrol IP group in the incidences of 53.3%, 33.3%, and 26.7%, respectively. Single and multiple adenomas was found in 13.3% (2/15) and 13.3% (2/15) of DMD alone group, 6.7% (1/15) and 0% in the resveratrol IG group and 6.7% (1/15) and 0% in resveratrol IP group, respectively. Adobe Photoshop CS5 software (Adobe Systems Incorporated, San Jose, CA, USA) was employed to measure lesion area accurately by the method described elsewhere [27]. The average area of lesions (hyperplasia/adenomas) of DMD alone group (0.599 ± 0.037 mm^2^) was 50.9% larger than that of resveratrol IG (0.397 ± 0.062 mm^2^) and 87.8% larger than resveratrol IP group (0.319 ± 0.040 mm^2^) with statistically significance (*p* < 0.05; Figure 3B,C).

### 3.4. Pathological Abnormalities in Other Organs

As shown in Table 2, the incidences of hepatocellularcarcinomas were 26.7%, 6.7%, and 6.7%, colon lymphadenosis were 46.7%, 26.7%, and 26.7% (*p* > 0.05) and lung fibrous hyperplasia were 20.0%, 13.3%, and 6.7% (*p* > 0.05) in DMD alone group, resveratrol IG group and resveratrol IP group, respectively. Those incidences between the DMD alone group and the two resveratrol treated groups were significantly different (*p* > 0.05).

### 3.5. Lower Serum Tg and CEA Levels in Resveratrol-Treated Rats

**Figure 4:**
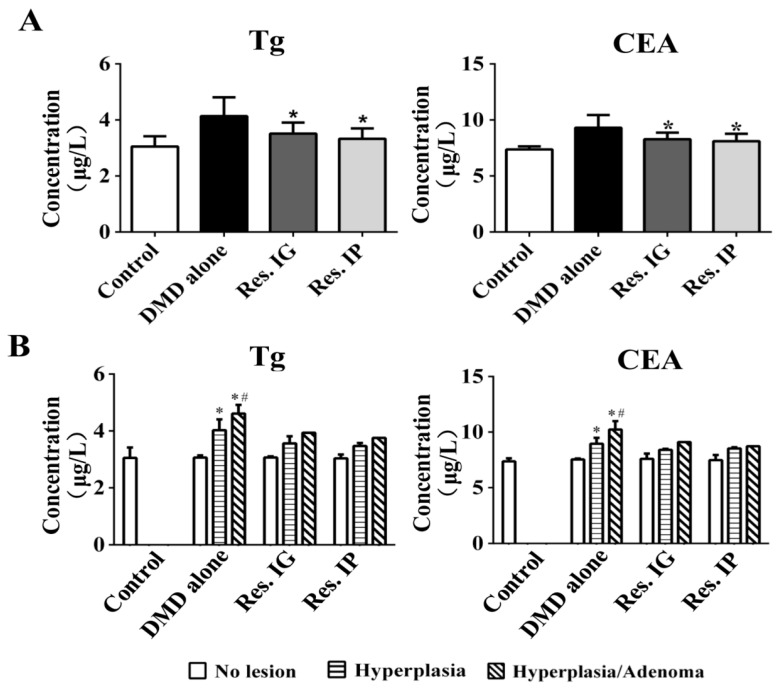
ELISA determination of Tg and CEA levels in rat serum. (**A**) The average serum Tg and CEA levels in the four experimental groups. *, *p* < 0.05 compared with DMD alone Group; (**B**) The average serum Tg and CEA levels in the rats of the same experimental groups without and with pathological lesions. *, *p* < 0.05 compared with lesion-free rats; #, *p* < 0.05 as compared with the rats with hyperplasia.

Tg (thyroglobulin) is elevated in the patients with thyroid nodules and adenomas, which is considered as a risk factor of thyroid cancers [28]. As shown in Figure 4A, the average serum Tg levels in resveratrol IP group (3.327 ± 0.304 μg/L) and resveratrol IG group (3.512 ± 0.377 μg/L) were lower than that of the DMD alone group (4.139 ± 0.628 μg/L; *p* < 0.05), but 9.0% and 15.0% higher than the control group (3.053 ± 0.365 μg/L; *p* > 0.05). CEA (carcinoembryonic antigen) is a well-known thyroid cancer biomarker [29]. The average serum CEA levels in resveratrol IP (8.111 ± 0.604 μg/L) and IG group (8.280 ± 0.541 μg/L) were lower than that of DMD alone group (9.306 ± 1.049 μg/L; *p* < 0.05) and 10.0% (*p* > 0.05) and 12.3% (*p* > 0.05) higher than the control group (7.373 ± 0.225 μg/L). In the DMD alone group, the CEA and Tg levels of adenoma-bearing rats were higher than that of the tumor-free rats (*p* < 0.05) as well as the rats with hyperplasia only (*p* < 0.05; Figure 4B). Within the individual experimental groups, the average levels of serum CEA but not Tg were relatively higher in the rats with lymphadenosis (*p* > 0.05; Supplementary Figure S1). 

### 3.6. Variable Levels of Growth-Related Factors in the Experimental Groups

**Figure 5:**
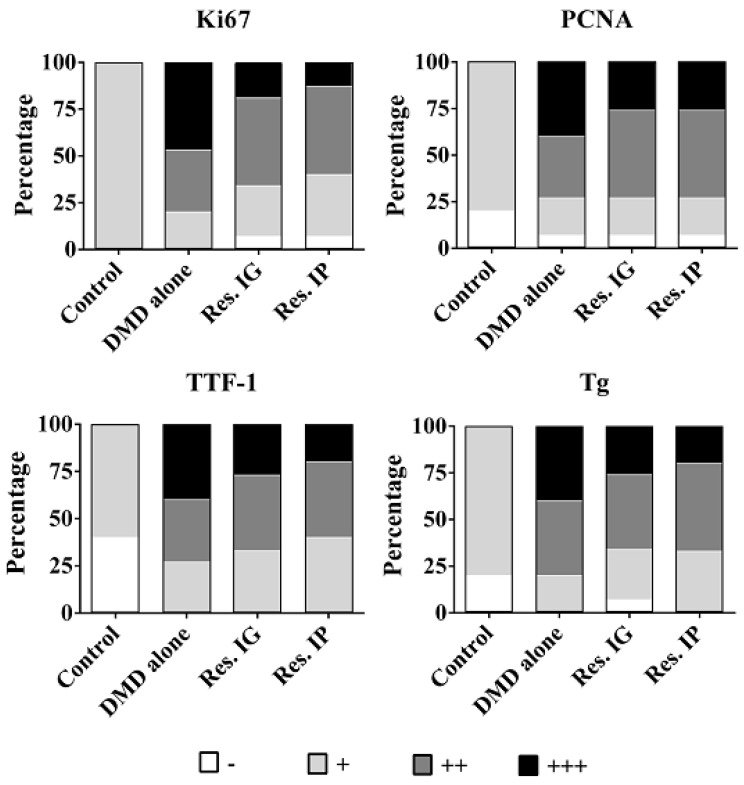
The fractionation of Ki67, PCNA, TTF-1 and Tg expression patterns in the thyroid tissues of the four experimental groups.

TTF-1 (thyroid transcription factor 1) is a homeobox transcription factor essential for the development of the thyroid, and plays a role in thyroid proliferation, migration, and tumorigenicity [30]. Ki67 and PCNA (proliferating cell nuclear antigen) are widely used in routine cancer investigation as proliferation promoters [31,32]. Immunohistochemical staining for TTF-1, Ki67, and PCNA were performed on rat thyroid specimens. As shown in Figure 5. TTF-1 was detected in the nuclei of thyroid epithelial cells of all groups and its labeling density in the specimens, especially the regions with pathological alterations, of the DMD alone group was stronger than that of other groups. Tg is expressed in thyroid specimens of the control group, which is elevated in the that of resveratrol-treated group and became more distinct DMD alone groups. The levels of Ki67 and PCNA in the DMD alone group were higher than that of the resveratrol-treated groups and their levels in the hyperplasia and adenoma tissues were stronger than the surrounding noncancerous tissues.

### 3.7. Resveratrol Inhibited NF-κB/p65 Signaling and IL-6 and COX-2 Expression

**Figure 6:**
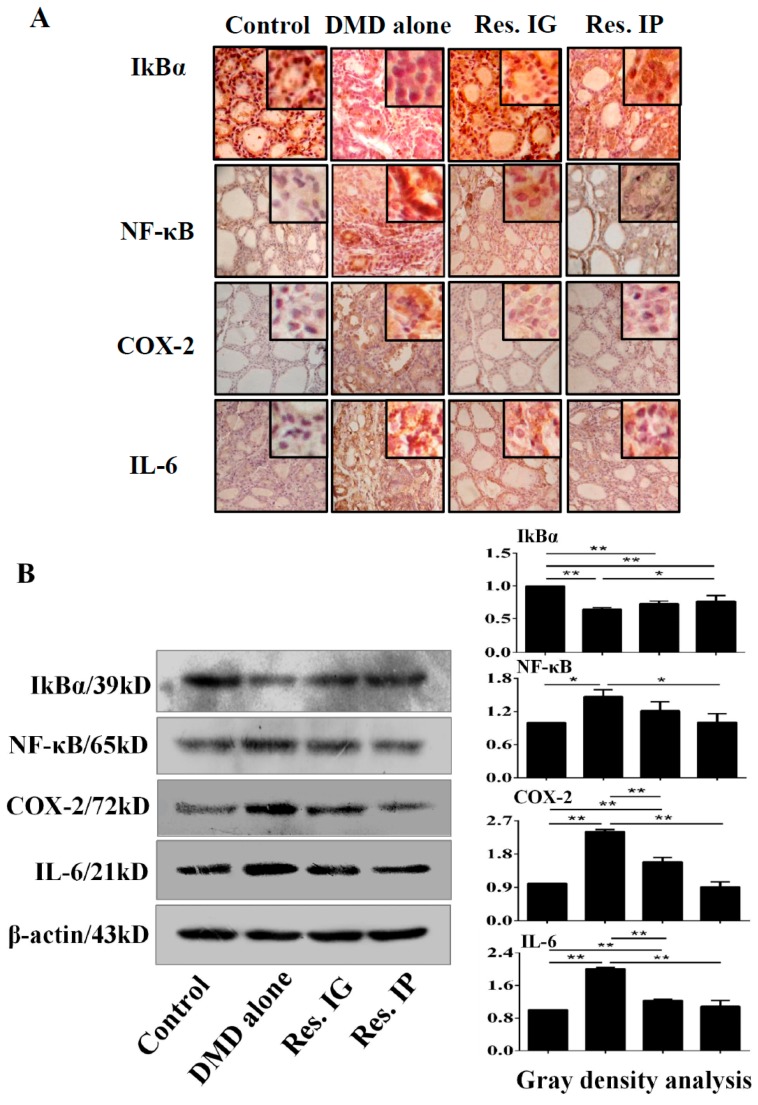
Effects of resveratrol on NF-κB signaling and its associated protein production. (**A**) IkBα, NF-κB, COX-2 and IL-6 immunohistochemical staining were performed on the Control, DMD alone, Res. IG and Res. IP groups; (**B**) Western blot analysis of IkBα, NF-κB, COX-2, and IL-6 expression in four groups. β-actin was used as quantitative control, and the results were statistically analyzed relative to control group. *, *p* < 0.05; **, *p* < 0.01.

NF-κB plays active role in inflammatory response and cancer initiation and progression. Normally, NF-κB is associated with IκBα and is retained in the cytoplasm inactively. Upon the stimulation by extracellular or intracellular signals, such as carcinogens, NF-κB is activated by proteasomal degradation of IkBα, leading to COX-2 and IL-6 expression [33]. The results of immunohistochemical staining (Figure 6A) and Western blotting (Figure 6B) showed that NF-κB/p65, IL-6, and COX-2 expression levels were relatively low in the control group and distinctly upregulated in the thyroid tissues of DMD alone group. NF-κB/p65, IL-6, and COX-2 levels in Res IG group and Res IP group were lower than that in DMD alone, but higher than that in the control group. IkBα was expressed in a high level in the thyroid tissues of the control group and became downregulated in the experimental groups, especially in the DMD alone one (Figure 6A,B).

## 4. Discussion

The incidence of thyroid cancer is dramatically increasing in the annual rate of 5.4% in men and 6.5% in women, and is expected to be the fourth commonest cancer by 2030 [34]. TC can be viewed as a gradually generated malignancy following the steps of initiation, promotion, and progression [35]. During the carcinogenic processes, genetic and epigenetic alterations, oxidative stress, chronic inflammation, and the activation of cancer-associated signaling pathways, leading to continues cell proliferation and finally malignant transformation [36]. Thyroiditis and thyroid nodule predispose to thyroid cancer [37,38,39], which are usually accompanied with activated NF-kB signaling [40,41] and the elevated serum levels of Tg and CEA [28,42]. The results of our current study are in agreement with the findings from the TC patients, because hyperplasia and adenoma(s) of rat thyroids are induced 30 weeks after DMD treatment, and the severity of the lesions are correlated with serum Tg and CEA levels. Moreover, the thyroid tissues of DMD-treated rats show TTF-1, PCNA, and Ki67 upregulation, suggesting their increased cell proliferation activity and the potential risk of malignant transformation [31,32]. Because the cancer-related pathological alterations are favorably occur in the thyroids (53.3%), rather than other major organs of the DEN/MNU/DHPN-treated rats, this cancer induction model would be more applicable to explore an agent that can suppress the cellular and molecular alterations in stepwise thyroid carcinogenesis. 

Resveratrol, a naturally polyphenolic agent, has a variety of beneficial biological effects, including cancer prevention and treatment [43]. This nontoxic compound has multiple molecular targets, including those that are involved in proliferation, survival, and death of cancer cells [44]. The in vitro inhibitory effects of resveratrol on thyroid cancers have been documented. For instance, resveratrol enhances the rates of ^131^I-induced cell death of thyroid cancer cell [45] and is able to suppress growth and to overcome retinoic acid resistance of human anaplastic thyroid cancer cells [13]. However, the in vivo impact(s)/influences of resveratrol in stepwise thyroid cancer formation is still lesser known. Because the preneoplastic lesions are successfully induced in the thyroids of the DEN/MNU/DHPN-treated rats, this model is employed to elucidate the preventive values of resveratrol in stepwise thyroid carcinogenesis by systemically administering resveratrol through IG and IP routes.

Resveratrol has been used as a dietary supplement or functional food because of its beneficial effects on health [12]. However, it has not yet been used for cancer prevention and treatment because of the uncertainty of its bioavailability in the major organs when administered systemically [46]. For this reason, the suitability of resveratrol in vivo application was evaluated by checking its concentrations in thyroid tissues 30 min after IP and IG administration in the dose of 20 mg/kg by weight before conducting further experiments. The results were compared with that of resveratrol-sensitive THJ-16T cells that were treated by 100 μM resveratrol for 60 min. It was revealed that cellular resveratrol uptake by THJ-16T cells was about 41.4% higher than the thyroid concentration of resveratrol administered by IG and almost equal (3.1% higher) to the concentration of resveratrol administered by IP route. Because 100 μM resveratrol is sufficient to causes growth arrest and apoptosis of THJ-16T cells, IG and, especially, IP administered resveratrol may exert certain biological effects on rat thyroid tissues although its actions may be relative mild than that in THJ-16T cells. Consequently, IG and IP administration pathways are applicable to rat thyroid carcinogenic model.

To elucidate thyroid cancer prevention potential of resveratrol, DEN/MNU/DHPN-treated rats were separated to untreated group as the control and the groups treated with resveratrol via IG and IP routes for 30 weeks, respectively. Histopathological examination revealed that the incidences of hyperplasia and adenomas of the DMD alone group are significantly higher than that of the two resveratrol treatment experimental groups. Moreover, those pathological alterations are more severe in the DMD alone group rats in terms of the extent of hyperplasia and the number and size of adenomas in the individual thyroid glands. Focal papillary hyperplasia is a common hyperplastic lesion of the thyroid and it continues proliferation may convert into adenoma and eventually carcinoma [47]. The pathological findings of current study reveal the potential of resveratrol to alleviate thyroid tissue lesions. It would be possible that resveratrol may delay the onset of thyroid tumorigenesis and therefore malignant transformation. Furthermore, the safety of long-term resveratrol administration is confirmed because all the rats of the two resveratrol-treated groups were kept alive during the 30 week resveratrol treatment, and their average body weights are higher than that of the untreated ones and without significant difference with the healthy rats.

CEA has been regarded as the general cancer-related protein [48] and elevated Tg levels are associated with thyroid carcinogenesis [29]. PCNA and Ki67 are localized in nuclei and are strongly associated with tumor cell proliferation and growth [49,50]. To further ascertain the inhibitory effects of resveratrol on DEN/MNU/DHPN-induced pathogenesis, the serum Tg and CEA levels and Ki67 and PCNA expression in thyroid tissues of the three experimental groups were checked and compared with that of the normal fed rats. It was found (1) that the average serum Tg and CEA levels of resveratrol IP and IG groups were higher than that of normal rats but significantly lower than that of the untreated group and (2) that their levels are in accordance with the severity of pathological lesions. Because the serum levels of CEA and Tg are not significantly different between the rats with and without patholigical alterations in other organs, their elevation would largely result from the thyroid abnormalities. Although TTF-1, Ki67, and PCNA are upregulated in the thyroid specimens of DMD-treated rats, especially in the regions with hyperplasia and adenoma formation, they are in relatively lower levels in the two resveratrol-treated groups. The above findings further demonstrate the effectiveness of resveratrol in attenuating the carcinogenic effects of DMD on rat thyroid tissues by suppressing proliferative activity, and, therefore, delaying the onset of tumor formation. Because the suppressive effects of IP and IG administered resveratrol have no significant statistical difference (*p* > 0.05), the IG approach would be more suitable for long-term application. 

Inflammation is a physiological process in response to tissue damage including the one caused by chemical carcinogens [51]. It is causally linked to carcinogenesis and acts as a driving force in premalignant and malignant transformation. NF-κB signaling pathway activated plays active roles in the inflammation processes. When phosphorylation-mediated IκB-α degradated, NF-κB activates gene transcription, such as COX-2 and IL-6 [52]. This case seems also holds true in thyroid cancer because NF-κB activation is frequently found in both thyroid cancers and the thyroid tissues with chronic inflammation [53]. Because anti-inflammation is one of the biological effects of resveratrol, the statuses of NF-κB signaling and the expression of IL-6, COX-2, and IκB-α in the thyroid tissues of the four experimental groups are elucidated. It is found that in comparison with the results that were obtained from DMD-alone group, the levels of NF-κB/p65, IL-6, and COX-2 expression in the thyroid tissues of the resveratrol treated groups are decrease, accompanied with the increased IκB-α expression. These finding thus provide additional evidence of the preventive effects of this polyphenol compound on thyroid tumor formation via suppressing DMD-caused and NF-κB/p65-mediated inflammatory reaction. 

## 5. Conclusions

Our study demonstrates that systemically administered resveratrol efficiently reduces the frequency and severity of thyroid cancer-related lesions in DEN/MNU/DHPN-induced carcinogenic model through inhibiting proliferation and suppressing NF-κB mediated inflammatory reaction. Long-term resveratrol administration improves the general health of DMD-treated rat and alleviates DMD-caused pathological lesions in the thyroids and other major organs. Oral administration can achieve similar therapeutic consequence as the intraperitoneal approach. In this context, this commercially available polyphenol compound would be favorable in the prevention of thyroid cancer.

## Figures and Tables

**Table 1:** The average weights (g) of animal body and major organs.

Treatment	No. of Rats		Body Weight			Relative Organ Weights			
	0 w	30 w	0 w	30 w	Thyroid	Liver	Lung	Kidneys	Spleen
Control	5	5	162.8 ± 5.4	610.1 ± 24.8	0.011 ± 0.000	2.49 ± 0.21	0.35 ± 0.03	0.59 ± 0.03	0.14 ± 0.01
DMD alone	15	15	162.6 ± 7.0	549.1 ± 42.1	0.013 ± 0.002	2.92 ± 0.65	0.36 ± 0.06	0.60 ± 0.06	0.14 ± 0.02
Res. IG	15	15	163.1 ± 7.7	580.5 ± 37.7 *	0.012 ± 0.002	2.73 ± 0.34	0.35 ± 0.04	0.62 ± 0.07	0.14 ± 0.01
Res. IP	15	15	163.9 ± 7.2	591.3 ± 38.4 *	0.012 ± 0.001	2.69 ± 0.39	0.35 ± 0.03	0.61 ± 0.07	0.14 ± 0.02

Data are expressed as Mean ± SD. *, *p* < 0.05 in comparison with the rats in DMD alone group.

**Table 2:** Incidences and severity of pathological alterations in three major organs.

Organs	Control	DMD Alone	Res. IG	Res. IP
	(*n* = 5) ^a^	(*n* = 15) ^a^	(*n* = 15) ^a^	(*n* = 15) ^a^
Liver carcinoma	0	4 (26.7) ^b^	1 (6.7) ^b^	1 (6.7) ^b^
Colon lymphadenosis	0	7 (46.7) ^b^	4 (26.7) ^b^	4 (26.7) ^b^
Lung fibrous hyperplasia	0	3 (20.0) ^b^	2 (13.3) ^b^	1 (6.7) ^b^

^a^ Number of rats; ^b^ Percentages in parentheses.

## Supplementary Materials

The following are available online at http://www.mdpi.com/2072-6643/10/3/279/s1, Figure S1: ELISA evaluation of serum Tg and CEA levels in the rats of the four experimental groups without and with pathological alterations in the liver, colon and lungs.
