# Supplementary material for: Preventive Potential of Resveratrol in Carcinogen-Induced Rat Thyroid Tumorigenesis
Source: Nutrients. 2018 Feb 28;10(3):279. doi: 10.3390/nu10030279 (PMC5872697; doi:10.3390/nu10030279)
Supplement: Supplementary file 1 [file nutrients-10-00279-s001.pdf]

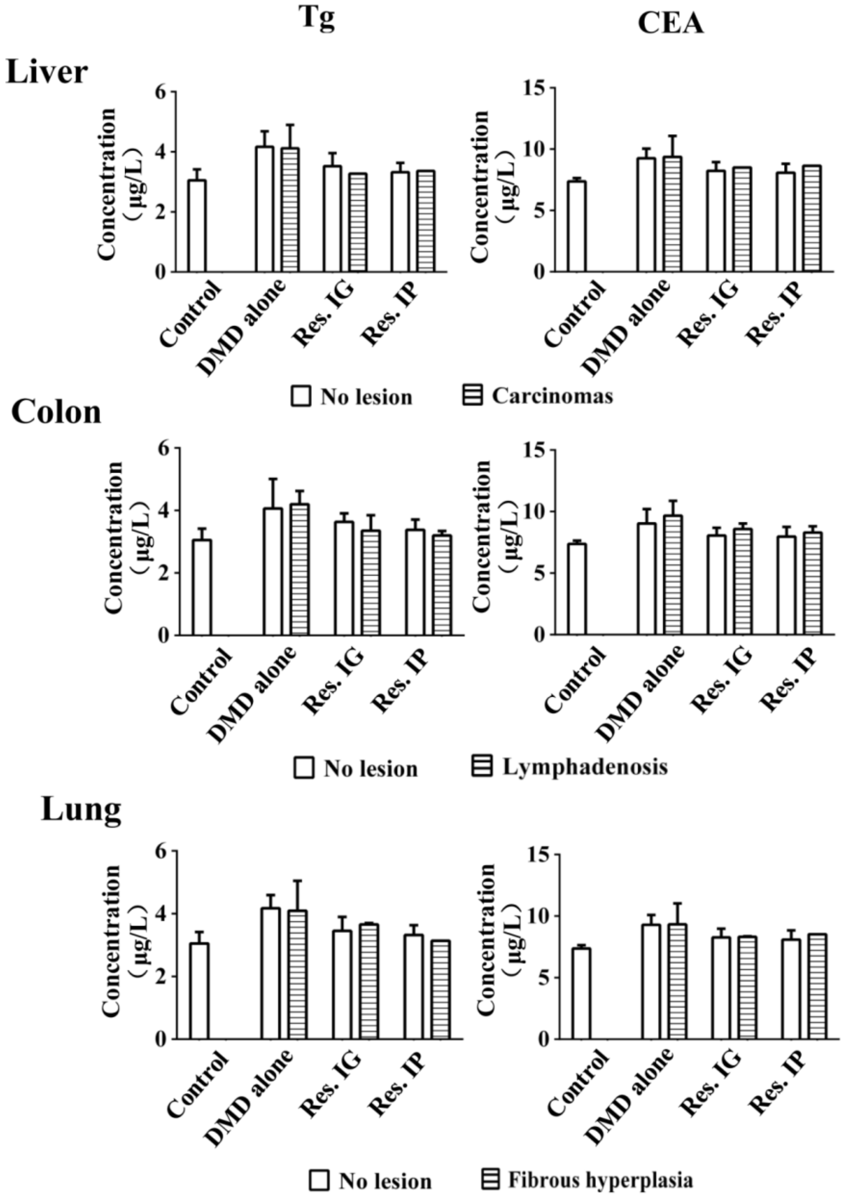

Figure S1: ELISA evaluation of serum Tg and CEA levels in the rats of the four experimental groups without and with pathological alterations in the liver, colon, and lungs.
